# Dynamic Load Balancing Strategy for Parallel Tumor Growth Simulations

**DOI:** 10.1515/jib-2018-0066

**Published:** 2019-02-14

**Authors:** Alberto G. Salguero, Antonio J. Tomeu-Hardasmal, Manuel I. Capel

**Affiliations:** University of Cádiz, Computer Science, Puerto Real, Spain; University of Cádiz, Computer Science, Puerto Real, Spain; University of Granada, Granada, Spain

**Keywords:** Cellular Automaton, High Performance Computing, Mathematical Oncology, Tumoral Growth Simulation, Parallel Programming

## Abstract

In this paper, we propose a parallel cellular automaton tumor growth model that includes load balancing of cells distribution among computational threads with the introduction of adjusting parameters. The obtained results show a fair reduction in execution time and improved speedup compared with the sequential tumor growth simulation program currently referenced in tumoral biology. The dynamic data structures of the model can be extended to address additional tumor growth characteristics such as angiogenesis and nutrient intake dependencies.

## 1 Introduction

Cancer is a well-known and studied disease. Actually, cancer is the common name given to a group of diseases related to the abnormal growth of cells. Dozens of cancers are already known, which may affect different organs of the body and whose study and treatment usually requires a bothersome medical examination or to go through painful invasive tests for the patient. The development of computer simulations makes possible to carry out certain experiments without the need for living tissue, thus reducing the cost and time of development, as well as avoiding some of the invasive tests.

*In silico* testing mainly refers to research conducted by computational methods or simulation and the ensuing results can be applied in the field of Biology or into the clinical medical actions. By establishing sound computer models of cell behavior, it will be possible to reduce costs and save precious time in terms of diagnosis and treatment following *in vivo* or *in vitro* testing. Computer models are currently able to produce relevant results and they do not generally present reproducibility issues when test measures must be repeated under exactly the same conditions. The Cellular Automata (CA) is a very suitable mathematical tool for the development of these computer simulations [1] since they can accurately simulate the behavior of each cell in tumors, according to its characteristics and the state of the environment. However, its usefulness is limited because current computer implementations of these simulations are very inefficient because of the immense number of cells and dynamic states that is necessary to represent.

We can find in the literature many proposals for improving the efficiency of CA-based simulations [2], [3], [4], [5]. All these proposals are based on an intelligent use of the different data structures and on the optimization of the instructions executed in the process, avoiding duplicate instructions as much as possible. However, all of them employ a sequential approach. Since none of them considers a parallel approach, most of the computing power of the current multicore processors is underused. The possibility of taking advantage of multicore and GPU parallelism as a promising multiplatform and framework to develop new programming techniques to speed up the simulation computation time has only just started to be explored [6], [7], [8], [9], [10]. In addition, the use of a parallel approach would allow the use of high-performance computing systems to further reduce the execution time of such simulations in the future.

This paper is an extension of the work we first proposed in [11]. In this paper, we evaluated a slightly modified version of the application that we previously developed, where some performance improvements have been made and some potential errors have been fixed. We have also extended the experiment to evaluate the impact on the efficiency of the simulation the frequency with which the load balancing between threads is applied.

Finally, we intend to apply the model and the presented overall parallelization technique to specific types of tumors such as prostate, breast or colon cancer. Our objective in future work is to investigate a feasible multiparadigm model which is capable of modelling the angiogenesis that is decisive for modelling cancer growth with metastasis (the growth inhibition induced by chemotaxis) and also the effect of therapies based on the presence of cytotoxic/cytostatic drugs.

## 2 Related Work

The use of the reticular cellular automata model for the simulation of self-organized biological systems or those with an organized emergent behavior is by no means new and is discussed in many publications [1]. The application of the specific model in tumor growth simulation has also been extensively studied in [12], [6], [13], [14], [15], [16], [4], [17] and [5].

Recent approaches to the parallel processing of cellular automata computation can meanwhile be found in the literature [18], [19], [20], [21], [7], [8], which support this type of processing both on a multicore processor or on manycore GPU architectures, mainly using CUDA-nVidia technology, and  on clusters. Most of the referenced authors use data parallelism by parallel processing different regions of an array that supports the state of the simulated tumor cells. Apart from a few exceptions, computer programs for cellular automata representation normally take the same number of threads as the number of available cores, and impose some synchronization condition, thereby obtaining speed-up values that are always greater than half the theoretical maximum possible.

In terms of tumor growth simulation, it is usual to take the sequential algorithm proposed in [14] as a reference, since it collects the minimal biological characteristics necessary to reasonably perform the simulation. On the basis of this algorithm, there are proposals that offer a finer model with the incorporation of biological characteristics such as tumor vascularization, nutrient gradient, etc [17]. The use of these models has an undoubted impact in tumor growth laboratory studies, thereby enabling a rapid and economic exploration of a wide variety of regions of the parametric space that the study of an *in vitro* tumor requires, limiting the regions of true interest of that space by means of computer simulation, and then passing to the *in vitro* study on these specific regions.

## 3 Background

There are various mathematical definitions of CA. We chose the one established in [22] as the most generally accepted definition in Computational Sciences, adapted to represent lattice-based biological models [12], [18], [19] and applied to simulate dynamic tumor growth in [14].

### 3.1 Cellular Automata

A CA is a 4-tuple ($\zeta,\varepsilon,N^{I},\rho$) where [23], [13], [14]:

- *ζ* is a discrete regular network of cells (also named nodes) together with certain border conditions which are set for the finite dimension network case and used to define neighboring conditions of cells on the network border. In our case, we have the mathematical representation of a 2D-plane: $\zeta=\{r:r=(r_{1},r_{2})\in Z^{2}\}$.
- *ε* is a finite set (usually with an algebraic Abelian ring structure) of states that the cell network can adopt.
- *N^I^* is a finite set of cells that define the neighboring cells with which a particular network cell can interact. $I\in\mathbb{N}$ is the maximum range, in any direction, a cell may affects another cell. Moore Neighborhood is obtained when *I* = 1.
- The transition function *ρ* that defines how a cell’s state can change over time and the state of its neighboring cells *N^I^*.

Given these definitions, any cell area can be defined as the network *ζ* included in the real 3D space *R^d^* that uniformly covers a portion of the d-dimensional Euclidean space. Each cell is labeled by its position $r\in\zeta$. Cell layout is spatially specified by the connections held by a cell with its closest neighbors and these are obtained by connecting pairs of cells following a regular pattern. For any spatial coordinate *r*, the neighborhood grid *N_b_*(*r*) consists of a list of neighboring cells that is defined by $N_{b}(r)=\{r+c_{i}:c_{i}\in N_{b},i=1,\cdots,b\}$, where *b* is the *coordination number* (i.e. the number of the grid-closest neighbors that directly interact with the cell located at coordinate *r*) and *N_b_* denotes any closest-neighbor pattern with elements $c_{i}\in R^{d}$, *i* = 1, ⋯, *b*. The total number of available cells is usually denoted by $|\zeta|$.

The entire set of neighboring cells whose states affects any cell *r* is defined by the *interaction vicinity* $N_{b}^{I}(r)$ function, which is defined by $N_{b}^{I}(r)=\{r+c_{i}:c_{i}\in N_{b}^{I}\}$. Although this neighborhood can be chosen in various different ways, for our simulation we have chosen Moore’s vicinity schema, where each cell only has its adjacent cells as neighbors. Furthermore, each cell $r\in\zeta$ has a state $s(r)\in\varepsilon=\{0,1\}$. A global configuration of the automaton $s\in\varepsilon^{|\zeta|}$ is determined by the state of all the cells on the grid.

Finally, the model’s temporal evolution dynamics is determined by the transition function *ρ* that specifies the changes in any cell state according to its previous state, and the interaction with its closest neighboring cells given by $\rho:\varepsilon^{\mu}\rightarrow\varepsilon$ where $\mu=|N_{b}^{I}|+1$, since the state of the current cell is also considered, in addition to the states of the neighbors.

The rule is proved to be spatially homogeneous and does not therefore explicitly depend on the position of a given cell. Extensions of the definition to include temporary or spatial homogeneity are feasible. If the CA is deterministic, the function of transition yields only one feasible change of state, whereas if it is stochastic, the new cell’s state is given by a specific distribution of probability according to the following equation:

(1) $$\rho(s_{N(r)})=\left\{\begin{array}[]{ll}z^{1}&\text{with probability}\ \ W(s_{N(r)}\rightarrow z^{1})\\ &\cdots\\ z^{|\varepsilon|}&\text{with probability}\ \ W(s_{N(r)}\rightarrow z^{|\varepsilon|})\end{array}\right.$$

where $z^{j}\in\varepsilon$ and $W(s_{N(r)}\rightarrow z^{j})$ is the transition probability to state *z^j^*, irrespective of time, given the vicinity configuration $s_{N(r)}$ .

### 3.2 Tumor Growth Simulation Models

One tumor cell in the model is an individual entity that takes up one node of a finite 2D lattice *ζ* and which can carry out the following actions [24]: migrate to another node on the grid, proliferate by mitosis, die or remain quiescent. A live cell can proliferate by generating a cell’s daughter through mitosis whenever there is a space available in its neighborhood (given by the Moore neighborhood). Our model tackles cell proliferation by applying a second distribution of probability, which is conditioned to the previous probability distribution and proliferation is given by a bi-valuated function $\rho_{p}(s_{N(v)})$. Consequently, the cell lattice position $v\in s_{(N(r))}$ will host the cell *r* resulting from mitosis if there is sufficient space. Finally, a live cell can migrate by changing its position within the tumor if there is free space to place it up on the lattice that simulates the tumor tissue. This situation has been tackled by using a third probability distribution which is conditioned to *ρ*_*l*_ and bi-valuated: $\rho_{m}(s_{N(v)})$.

The ACT algorithm is derived from the previous analysis1We have presented the algorithm in pseudocode but in order to carry out the Java implementation, the principles of structured programming have been followed. which amounts to a sequential simulation of the standard well-known tumor growth model. The model is parameterized by the probability distributions described in the previous section, which enables us to use a simulation based on a Monte Carlo stochastic submodel. The proposed model decides whether a cell dies, remains quiescent or proliferates by mitosis (if there is enough space around the cell to do so).

Having established whether a cell can proliferate, the *proliferation* routine in line 13 subsequently decides whether there is room for the new resulting cell mitosis and then places a new active cell in quiescent phase during the cell position update routine.


Algorihtm ACT
	Input:
	nGen, nCells, cells[x][y],
	W(s_N(r)->z_1 ), W'(s_N(v)->z_1 ), W''(s_N(v)->1).
	Output: Tumor growth in cells simulation.
	1. Assign initial tumor-seed in cells[x][y];
	2. For(i=0; i<nGen; i++)
	3. For(x=0; j<nCells; j++)
	4. For(y=0; y<nCells; y++)
	5. rr<-random( );
	6. If(rr >= W(s_N(r)->z_1 ))
	7.  cells[x][y]=0;
	8.  goto(line 2);
	9. If(rr < W'(s_N(v)->1))	
	10. PH++;
	11. If(PH >= NP)	
	12.  Pi<-random(); i=1, 2, 3, 4.
	13.  If(proliferation()) GoTo (line 4);
	14.  ElseGoTo (line 2);
	15. Else
	16. rrm<-random();
	17. If(rrm < W''(s_N(v)->1))
	18.  Mi<-random(); i=1, 2, 3, 4.
	19.  If(migration()) GoTo (line 4)
	20.  ElseGoTo (line 2);
	21. ReupdateCell Positions;
	22. GoTo (line 1);

(2) $$\rho_{p}(s_{N(v)})=\left\{\begin{array}[]{@{}ll}1&\text{with probability}\ \ W^{\prime}(s_{N(v)}\rightarrow 1)\\ &\cdots\\ 0&\text{with probability}\ \ W^{\prime}(s_{N(v)}\rightarrow 0)\end{array}\right.$$

(3) $$\rho_{m}(s_{N(v)})=\left\{\begin{array}[]{@{}ll}1&\text{with probability}\ \ W^{\prime\prime}(s_{N(v)}\rightarrow 1)\\ &\cdots\\ 0&\text{with probability}\ \ W^{\prime\prime}(s_{N(v)}\rightarrow 0)\end{array}\right.$$

This is performed by determining the actual spaces in a cell’s neighborhood and by migrating cells into these. The absence or presence of spaces is provided by a probability function. If mitosis is not possible for this cell since no space is available then analysis of cell division feasibility will be passed on to the next cell. The cell migration phenomenon is simulated in lines 16–20 of the algorithm following a similar approach, and the probabilities for determining cell migration direction are also decided. Cell migration occurs if there is space for migration with no other effect. Taking this as the first approach to the concurrent tumor growth simulation model, we then developed a program that was the initial sequential simulation of cell growth with a single thread. The initial simulation was configured by assuming an initial state of four tumor-cells and two hundred cycles as the total run time for a cell lattice comprising 32 × 32 cells by assuming the model control characteristics shown in Table 1. This table displays the described set of probabilities that parameterizes our stochastic model; whereas variable NP represents how many signals a cell requires to proliferate (only one in this initial approach,) and then exit the *G*_0_ phase of the cellular living cycle. The *G*_0_ phase of the cell cycle is that phase of the cycle where the cell is quiescent from the mitotic point of view and it is not divided. That is, the cell is dedicated to its nutrition, the protein synthesis and the excretion of metabolic waste. Variable PH defines the number of proliferation signals.

**Table 1:** Parameters of the tumor simulation model.

Simulation Parameters	Values
$W(s_{N(r)}\rightarrow 1)$	0.8
$W(s_{N(r)}\rightarrow 0)$	0.2
$W^{\prime}(s_{N(v)}\rightarrow 1)$	0.2
$W^{\prime\prime}(s_{N(r)}\rightarrow 1)$	0.25
PH (no. proliferation signals)	1
NP (signals to proliferate)	1

**Figure 1:**
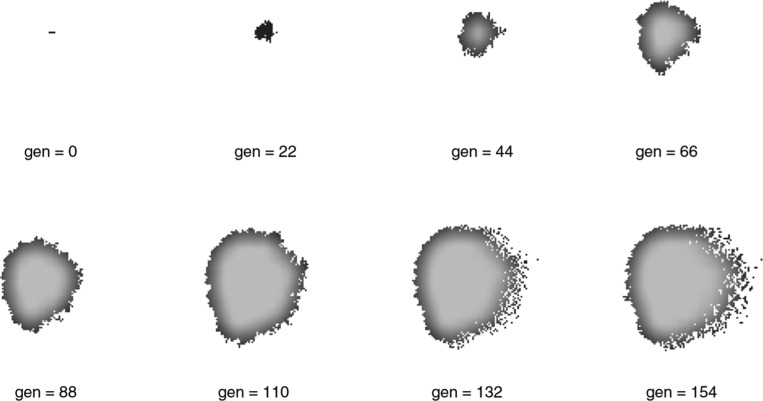
Evolution of a tumor model simulation assuming the *parametric load* shown in Table 1 for a tissue domain of 128 × 128 cells and 154 generations.

The ACT algorithm with the parameters in Table 1 produces the graphic sequence of snapshots displayed in Figure 1 that shows simulated tumor evolution. These parameter values guarantee fast growth of the tumor that invades the surrounding tissue in a dense and compact way as described in reference [25]. Obviously, simulations of this size have too few cells or generations to be useful in practice. In a previous work [25], the authors state that in the case of tumors with well-known growth dynamics, such as breast or prostate ones, the tumor must have at least 10^9^ cells to be clinically detectable, which is equivalent to 1 g wet weight. Evolution may take between 2 and 8 years to reach this point of tumor evolution depending on the aggressiveness of the tumor cells according to their histological type. In order to perform simulations that are closer to the actual dynamics of a real tumor, we must therefore handle tissue *arrays* of at least $10^{5}\times 10^{5}$ cells. In practice, implementation of such simulations must include between 17,520 and 70,080 steps of discrete time. By way of example, a simulation of $10^{3}\times 10^{3}$ cells running over 5000 steps of discrete time (roughly equivalent to 200 days of pathology evolution) requires 133 s of execution time with an Intel^©^ i5-4440 (3.10 GHZ) if single-thread simulation implementation is used.

## 4 Parallelization of the Tumor Growth Model

In this section we propose a generic parallel model for the simulation of tumor growth.

A brute force parallelization of the Polesczuk-Enderling sequential algorithm [2], whereby each thread evaluates a subset of the cells in the lattice, yields a poorly efficient algorithm because every access to the lattice must be carried out in mutual exclusion.2The original source code for the Polesczuk-Enderling algorithm can be found at http://jpoleszczuk.pl/?p=85. A modified version where all the debugging statements have been removed and statistic information is collected has been released along with the source code developed in this work.

**Figure 2:**
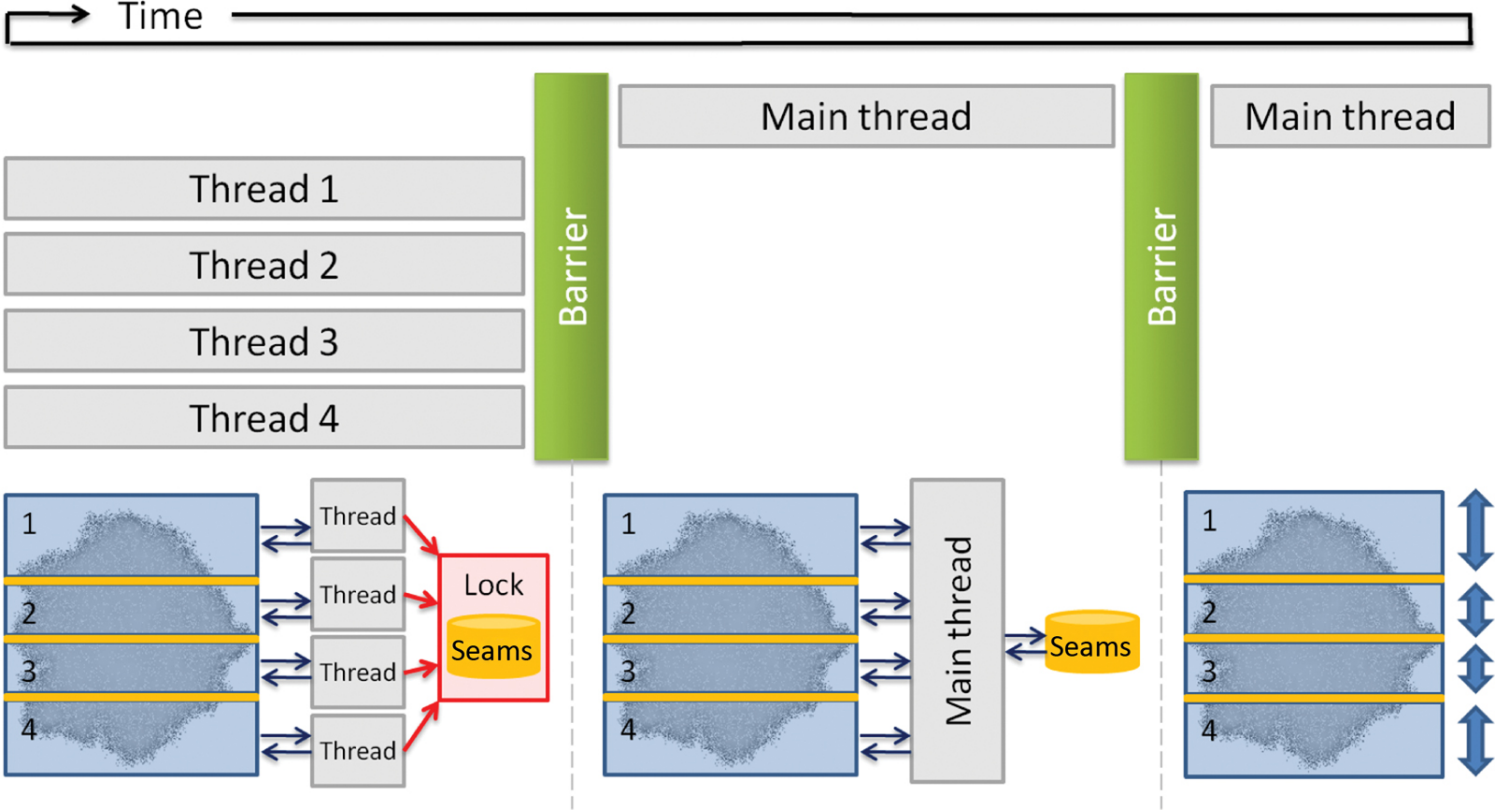
Tumoral growth parallel simulation.

The strategy proposed here for solving the parallelization problem relies on the creation of different lattice areas which are handled by a single thread exclusively. There is, however, one problem with the borders of these areas: two threads may write to the same cell, thereby violating mutual exclusion. For this reason, between non-blocking cell areas, it is necessary to define certain shared areas which are only accessed by threads under mutual exclusion (see Figure 2). Only one global shared area is in fact required between two adjacent non-blocking areas.

As we mentioned previously, tumor growth is simulated by cell evaluation through a discrete series of temporal states. It is necessary to evaluate each tumor cell individually in each of these cell states or *simulation steps*.

One widely used mathematical tumor growth model was outlined by Poleszczuk-Enderling [2] who implemented the model as a sequential C program. The approach is based on a sequential discrete cellular tumor growth automaton model.

The parallelization strategy usually followed for speeding up such a tumor growth simulation program consists in splitting the cell lattice assumed by the cellular automaton into different areas and processing each one with a different *thread* or *core*, thereby avoiding data races. However, this brute force parallelization of this simulation model has two drawbacks which result in low-performance algorithm implementations:

1. There is a great deal of unnecessary computation since every cell in the cellular automaton lattice is processed in each simulation step, but a large number of cells do not change their state because they are still far from a tumoral mass of appreciable size.
2. Cell-state processing is affected by a certain contention ratio because of the mutually exclusive access of concurrent execution threads to the cell lattice.

In order to address the first issue, we can assume a dynamic tumor growth model of the lattice. However, in this case, a large number of cells are still unnecessarily evaluated since the tumor growth mass pattern is a circle and the cell lattice grows following a rectangular shape in the computational model.

According to Poleszczuk-Enderling’s model [2] for cellular automata-based tumor growth, the most efficient way of processing tumoral cells consists in keeping a linear list of the tumor cells in the lattice that occupy sequential positions in memory. The list is entirely processed in each simulation step and this yields a new list of cells to be processed in the next one and so on.

The lattice is used here merely to keep the current state of the tumor. Only the changes in cells are actually written to it. Only two lists are in fact needed to implement the tumoral growth simulation procedure: one listing the cells still to be processed and the second one storing the tumor cells for the next simulation step. This scheme is similar to the one proposed by Polesczuk and Enderling [2], which is, to the best of our knowledge, the best sequential solution to the tumor growth simulation problem to date. However, our model uses multicore processors to improve the speed of the tumor growth simulation.

In order to speed up the tumor growth simulation model, two fundamental problems must be solved: firstly, to find a good strategy to maintain balanced cell distribution among threads; and secondly, to prevent blocking accesses to the data structures (the lattice and lists of active cells) as much as possible. It is, however, important to note that insertions and deletions on the lattice cell lists must be performed under mutual exclusion to avoid loss of information.

By using different lists for each region it is possible to prevent blocking access to the *current-state* cell list. The *next-state* lists must always be accessed by using blocking access primitives, since cells can migrate among regions when program threads access such lists. 

**Figure 3:**
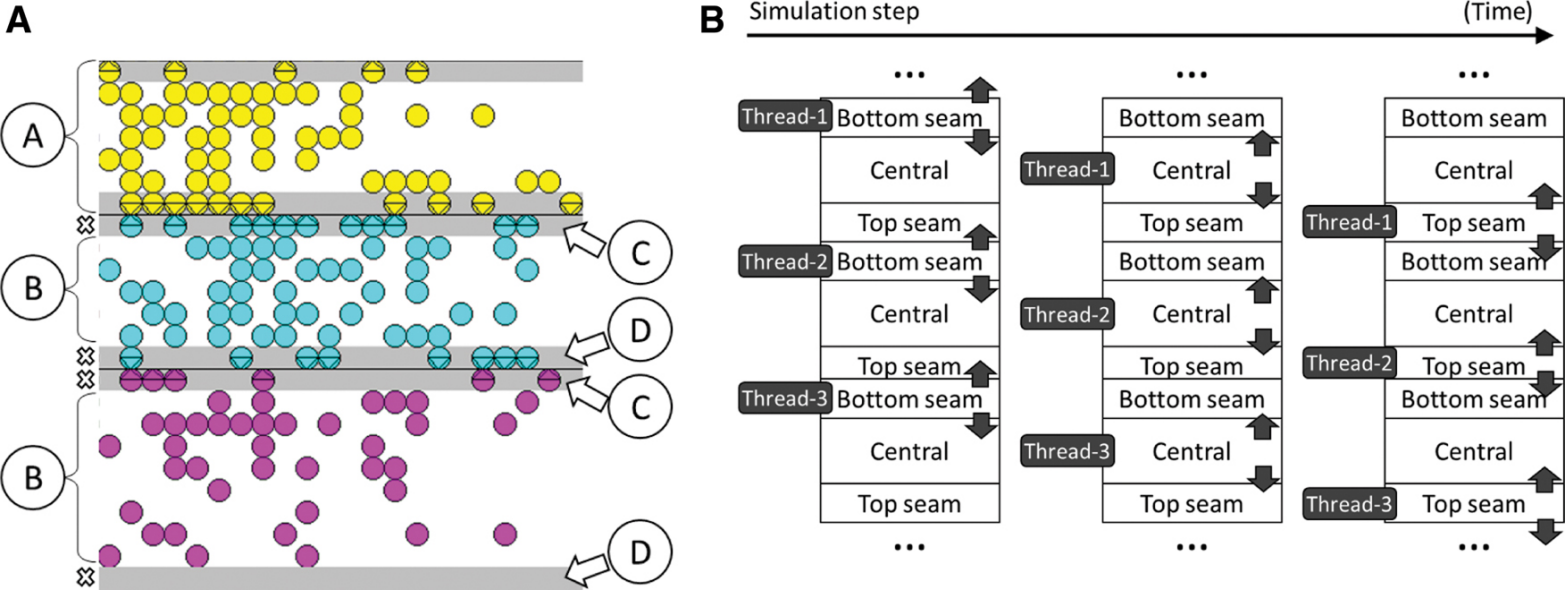
Tumor growth evolution model (A) Lattice division, (B) A step in the parallel simulation.

The main objective of the model proposed in this work is to enable the concurrent processing of the cells without the need of using blocking access mechanism for accessing both the lists of cells and the lattice that holds the current state of the tumor mass. To achieve this goal, the lattice is first divided vertically into as many regions (A) as threads available, as shown in Figure 3A. Each of these regions is further divided into three parts or subregions: top seam part (D), central part (B) and bottom seam part (C). The central parts of the regions are therefore separated by what we call ”seams”, which are divided into two parts. This layout guarantees the existence of at least two types of subregions between subregions of the same type. In other words, there are at least two subregions of a seam type (top and bottom) between two central parts, for example. There is also a central part and a top seam part between two consecutive bottom seam subregions, and the same occurs for the top seam parts. This allows the model to concurrently process the cells in subregions of the same type without needing to block access since threads do not enter subregions which are being accessed by other threads. In the case of cell migration to another region, no other thread may access the target subregion at the same time. In order to make use of this property, the proposed model first processes the cells in the bottom seam subregions, then processes those in the central parts and finally those in the top seam subregions (see Figure 3B). All this processing is carried out concurrently and without blocking the threads, except for making them wait until the remaining threads have processed the same type of subregion.

For the model to work efficiently, it is important to perform a correct load balancing between the number of cells that each thread obtains for processing. The load balancing depends on how the different regions are initially laid out and how these regions are re-assigned to threads during simulation. Once the entire next state of the tumor has been calculated, the main thread is responsible for adjusting the regions, if appropriate.

Subregions must have a minimum height so that no single cell can migrate among non-adjacent subregions. In the proposed model, the minimum height value is *δ*, the length of the longest displacement of a cell in a simulation step.

### 4.1 Initial Seams Adjustment

Tumor growth simulations usually start from one or several tumor cells around the center of the lattice. The tumor grows in a roughly circular shape. In order to achieve a uniform distribution of cells among threads, it is first necessary to calculate the dimensions of the initial tumor mass. Since the regions divide the lattice vertically, we only need to know its initial height, *H*^0^ = row_max_–row_min_. It is a common occurrence that the initial height of the tumor mass is not large enough to be divided into as many regions as threads available. In such a case, the minimum height to accommodate such a number of regions is used, i.e. $H^{0}=3\delta t$, where *t* is the number of threads.

In order to achieve maximum efficiency, the proposed model distributes the seams in such a way that there are equal numbers of regions above and below the center of the initial tumor mass. This layout guarantees that cells fill up all regions as soon as possible. As Figure 3A shows, the first and the last seams always start and end at the first and last rows of the lattice, respectively. Therefore, the first region always starts from the first row of the lattice and the last region always ends at the last row of the lattice.

### 4.2 Seams Adjustment During Simulation

**Figure 4:**
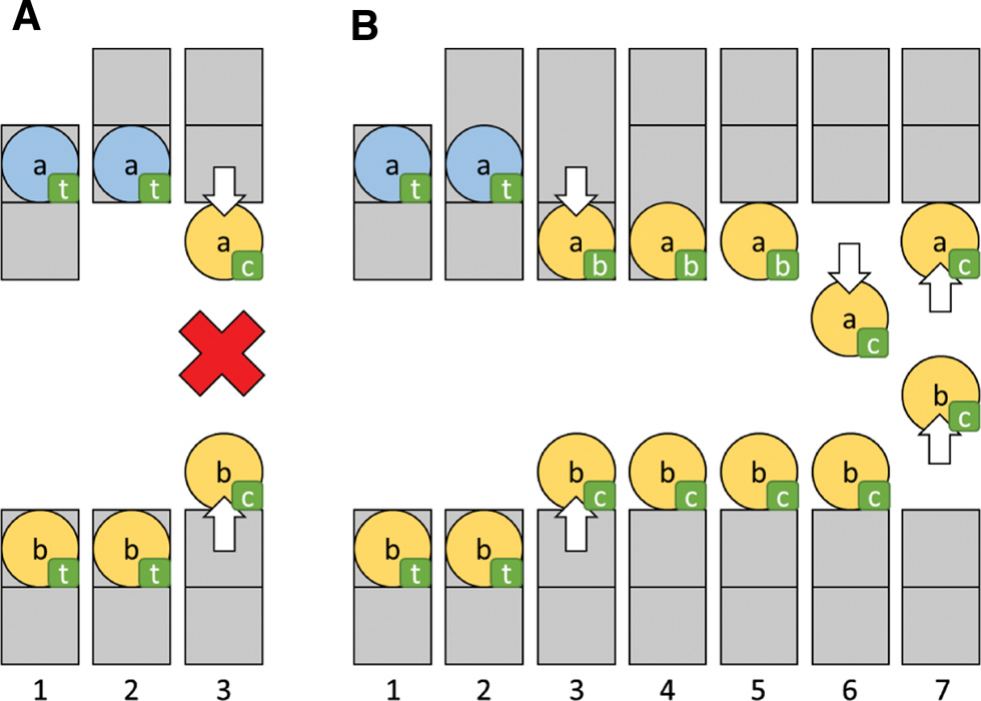
(A) Incorrect and (B) correct parallel seam adjustment. The small letters in rectangles indicate the current list the cells belong to (t = top seam part, b = bottom seam part, c = center).

As the tumor grows, it is necessary to adjust the seams to equally distribute cells among all the threads. The main thread is responsible for performing this task once the remaining threads have finished calculating the next state of the simulation. For reasons of efficiency, the main thread does not update the subregion to which each cell belongs. When the cells are again evaluated by the threads in the next step, the cells are assigned to the corresponding subregion. However, this may cause a cell to directly migrate from the top part of a seam (t) in one region to the central part of another region without passing through the bottom seam subregion, as shown in Figure 4A. In substep 2, when the upper seam has been moved upward, the cell still remains in the list of the top seam part (t). In substep 3, both threads simultaneously process the top seam parts (t) associated with the lists of cells, and this may cause incorrect concurrent access to the list of cells in the center area (c) or the need to use blocking access primitives. To avoid this situation, the seams are displaced in a three-tier process that is performed in three3For the sake of simplicity, only substeps where changes have been made to the lists are shown in Figure 4. Substeps 3 and 4 are actually part of the same overall step.  consecutive steps of the simulation, as shown in Figure 4B:

1. Increase the thickness of the seam in *δ* rows in the same direction of the displacement (step 2 in Figure 4B).
2. Decrease and increase the size of the subregions that share the seam in *δ* rows: the part of the seam that was increased in the previous step is decreased and the other part is increased, maintaining the same seam thickness (step 4 in Figure 4B).
3. Decrease the thickness of the seam to the default value in the region that has increased its size (step 5 in Figure 4B).

In this case, cell *a* can be safely added to list (c) in substep 6 because cell *b* cannot be processed in the same substep (they are still in different subregions). Since both cells have been assigned to the same thread in substep 3, they will not be processed concurrently. For this reason, there is no need to use blocking access primitives in substep 7, when both cells modify the center list (c).

The seam adjusting process is automatically started when a significant difference of cells between two adjacent regions is detected. An experiment has been performed in order to find the optimum threshold for performing the seams adjustment. The results are shown in Section 5.1.

It should also be noted that for reasons of efficiency it is not possible to adjust two consecutive seams. Since the main thread does not reassign migrated cells, it is not possible to determine the number of remaining cells in affected regions. Although this value could easily be calculated, it is important to note that the remaining threads are blocked until the main thread adjusts the seams, so it is crucial for seam adjustment to be performed as quickly as possible.

## 5 Experiment

In order to verify the efficiency of the solution proposed in this work, a series of experiments have been conducted. In these experiments we measured the time taken by a Java application to follow the proposed model4The source code of the application has been released under the GPL open source license at https://sourceforge.net/p/parallel-tumoral-growth/. and we compared the results with the best known implementation to date [2]. It is not possible, however, to make a direct comparison between two different executions of any of these applications. Since the final result in the concurrent version depends on the order in which the cells are evaluated by the threads, it is never possible to obtain the exactly same tumor as result when using multiple threads. This is because the order in which cells are evaluated matters, but we cannot control the execution speed of the threads. For this reason, in order to compare different application configurations, the number of cells processed per second has been taken as reference. This is still not a perfect measure, since the final tumor size has some impact in the performance of the simulation. Cells at the border of the tumor, with higher number of surrounding empty spaces, require more time to be computed. The bigger the tumor mass, the higher the number of cells in the border. For the same reason, this measure is also impacted by the density of the tumor mass. A tumor mass with a very diffuse border (see gen = 154 in Figure 1) requires more time to be computed than another one with a sharp edge. In order to minimize these effects, we have always repeated the simulations five times for each configuration and the average values have been taken as results.

All the simulations of the experiment have been run on an Intel (R) Core i7-7700HQ (2.8 GHz, 4 cores, 8 threads) and consisted of depositing a tumor cell in the center of the grid and performing four thousand simulation steps. The number of cells of the resulting tumors has varied between 103,704 and 133,964. The total number of cells processed in the simulations has varied between 115,556,397 and 159,823,438.

Actually, two different experiments have been performed in this study. The first experiment tries to determine how often the load of the threads has to be balanced. The second experiment evaluates the impact of the number of threads employed to solve the problem.

### 5.1 Seams Adjustment

The first experiment we have developed in this work aims to determine how often the load of the threads should be balanced. To do this, we have added to the implementation a procedure that automatically determines when load balancing must be performed between two threads that process adjacent regions. This procedure is based on the relative difference between the number of cells in the regions. When the relative difference between two adjacent regions, expressed as a percentage, exceeds a certain threshold (*ρ*), the procedure described in Section 4.2 is applied to balance the number of cells in the two regions.

**Figure 5:**
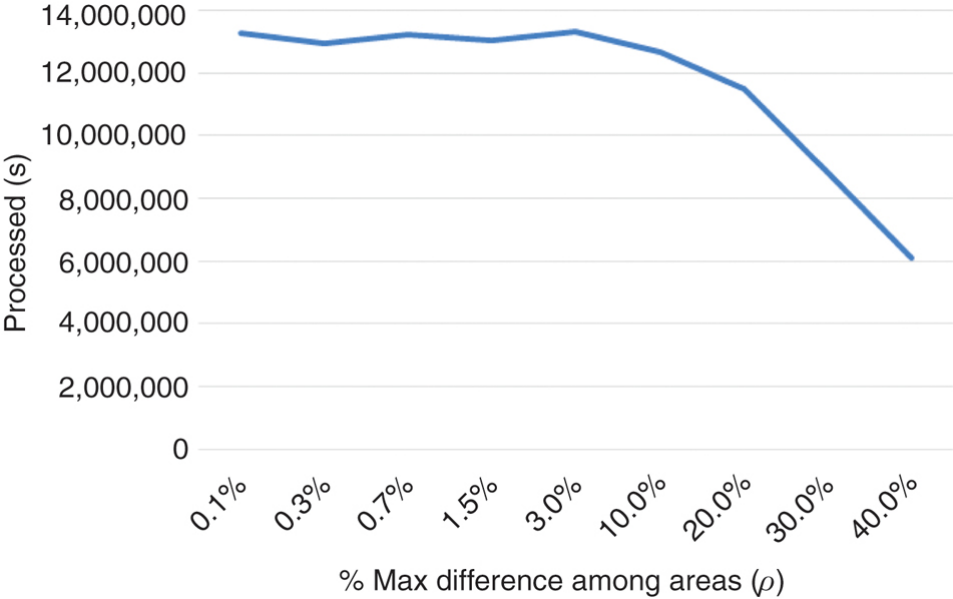
Number of cells processed per second with respect to *ρ*.

**Figure 6:**
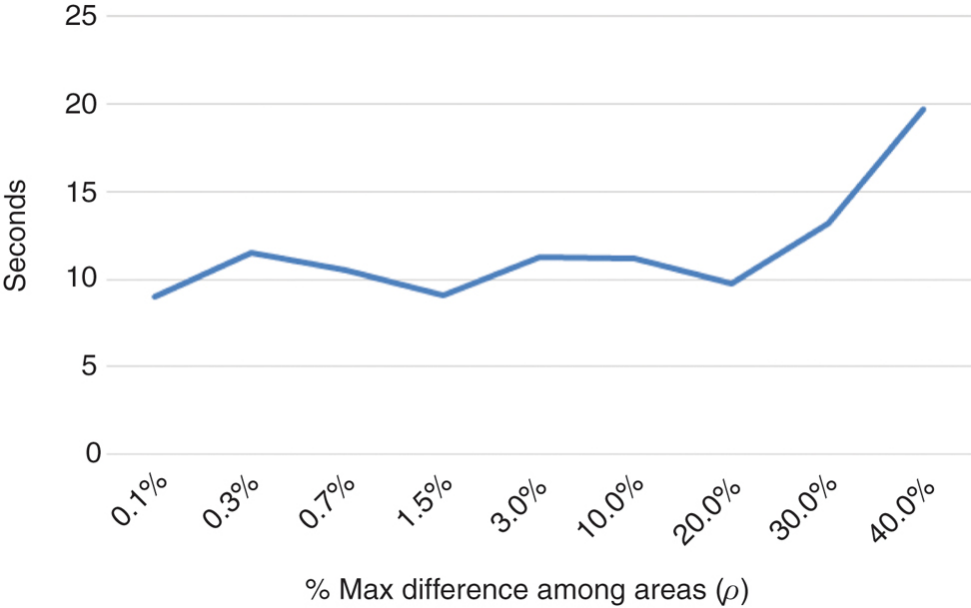
Total time with respect to *ρ*.

The experiment began trying to find out the approximate number of threads that produces the best performance. For this, a value of *ρ* = 10% was assumed and multiple simulations were launched, varying the number of threads between 2 and 30 ($n=2,4,\ldots,30$). The best result was obtained for *n* = 14. For this number of threads the experiment was repeated, but the rho value varied between 0.1% and 40% this time. The time spent for each value of *ρ* and the number of cells processed per second are shown in Figure 5 and Figure 6, respectively. As can be seen, there is a significant degradation of the performance for $\rho > 20\%$. For $\rho < 20\%$ the performance of the simulation is very similar. No significant differences are observed. In fact, this is because for $\rho < 20\%$ the load balancing process is performed almost for each simulation step. Especially when the number of cells is high. Moving a seam one row may involve the migration of hundreds or even thousands of cells from one region to another. The variation can be even more noticeable if we take into account that the adjustment of a seam requires four simulation steps, as described in Section 4.2. The number of cells in a region can vary greatly in each simulation step, without the need of seams adjustment. In fact, the number of cells in the border regions grows much faster than that of the central regions because they contains more cells at the border of the tumor.

In view of the results we can say that it is convenient to perform the load balance as often as possible. The time that the threads must pass blocked while waiting for the main thread to adjust the seams is compensated by the reduction of the waiting time in the barrier that synchronizes the threads.

### 5.2 Speedup

**Figure 7:**
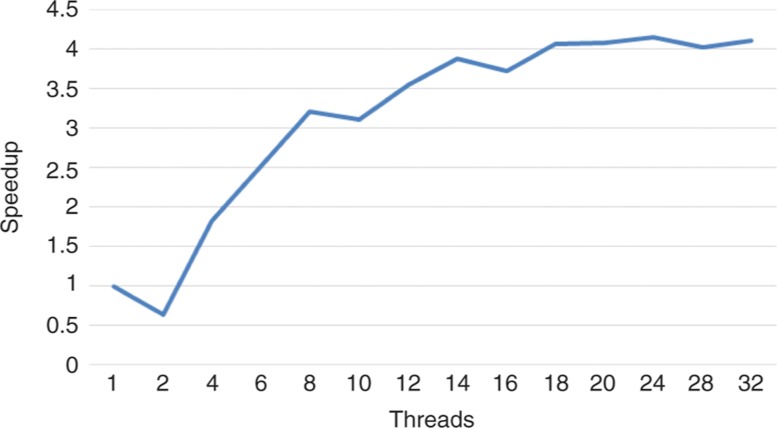
Performance (measured in cells processed per second) gain with respect to the best known sequential implementation.

**Figure 8:**
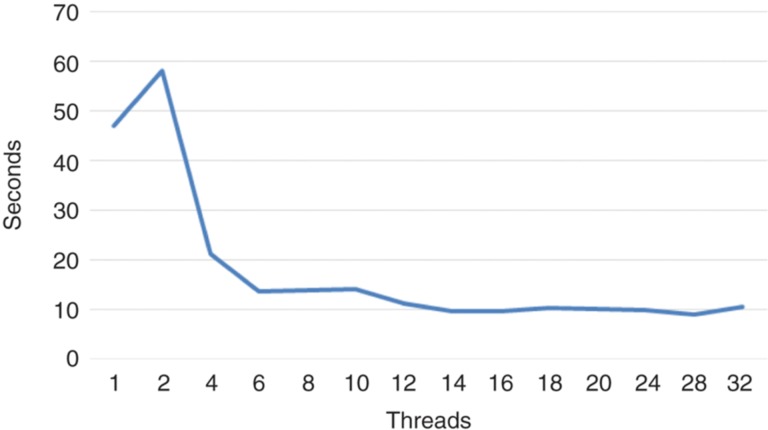
Total time with respect to the number of threads.

The objective of this experiment is to determine the optimal number of threads to be used when performing the simulation. For this, we have carried out several simulations with the same configuration parameters, but varying the number of threads between 2 and 32. The results obtained can be seen in Figure 7 and Figure 8. More detailed results can be found in Table 2.

In view of the results, we can confirm that the procedure presented in this work obtains better results than the best implementation available up to date. The time improvement is very significant: the sequential version requires 47.03 s to simulate 4000 simulation steps, while, in the best case, the multi-threaded version only requires 8.98 s for completing the same task. The sequential version is capable of processing 3,398,185 cells per second whereas the multi-threaded version is capable of processing up to 14,126,253 cells in the same time period of time.

**Table 2:** Simulation performance with respect to the number of threads (*ρ* = 0.01). The single thread simulation corresponds to the sequential implementation proposed in [2]. Speedup is calculated with respect to the number of cells processed per second.

Threads	Final size	Processed	Processed/s	Seconds	Speedup
1	133,964	159,823,438	3,398,185	47.03	1
2	111,134	124,198,942	2,195,565	57.99	0.65
4	112,523	127,172,530	6,203,735	21.16	1.83
6	103,704	115,556,397	8,587,091	13.72	2.53
8	128,363	150,704,897	10,927,714	13.82	3.22
10	126,278	148,331,796	10,567,864	14.20	3.11
12	120,164	135,795,797	12,081,374	11.25	3.56
14	111,677	128,189,958	13,189,048	9.74	3.88
16	109,444	124,148,561	12,652,588	9.58	3.72
18	122,429	141,957,555	13,803,220	10.29	4.06
20	120,613	139,303,150	13,894,192	10.01	4.09
24	119,695	139,888,658	14,126,253	9.90	4.16
28	110,539	124,156,688	13,671,410	8.99	4.02
32	125,999	146,534,049	13,967,509	10.49	4.11

With regard to the number of threads used, we can say that there is an improvement in performance as the number of threads is increased. The improvement in performance is more noticeable until the number of processor cores is reached (8 logic cores). This makes sense, because the procedure proposed in this work is designed to avoid the locks among threads as much as possible. The improvement of the performance is less noticeable from that number of threads, being almost null from fourteen threads. The degradation in performance with respect to the Polesczuk-Enderling version when only two threads are used is also noteworthy. In this case, the parallel processing of the two regions does not compensate for the work that the main thread has to do to balance the workload.

It is also worth to note that the brute-force parallel approach, where the threads employ blocking access mechanisms to modify the lattice and the lists of cells, has a really low performance, as expected. It requires more than 3 min to perform the four thousand simulation steps, whereas the method proposed in this work barely needs 10 s. This is because the brute-force approach has to perform almost a million blocking accesses when performing the simulation, degrading its performance.

## 6 Conclusions

Tumoral biology research is a very active research area that aims to develop more effective and less toxic treatments for patients. Mathematical modeling and computational simulation applied to tumoral biology are considered to be valuable auxiliary disciplines since they are more cost-effective and use fewer human and material resources than a research laboratory. *In vivo* or *in vitro* experiments are now conducted through mathematical models and it is only necessary to consider parameters that are relevant for the tumor growth and rule out any non-useful results that have previously identified by simulation.

The basic algorithm and its improvements for performing tumor growth simulations with cellular automata were established quite some time ago and they have been widely used for programming computer tumor growth simulations ever since. However, their utility is limited because they usually employ a sequential approach to implement the cellular automata-based tumor growth simulations, which reduces their efficiency in modern computer systems. In this paper we have proposed a parallelization of this well-known algorithm for the tumor growth simulations with cellular automata. The best sequential implementation that we have found in the literature [2] manages to accelerate the simulation algorithm by ingeniously optimizing the cellular automaton data structures. The proposed parallel simulation scheme employs data parallelism with load balancing and one barrier condition for thread re-synchronization. The Java thread pool executor is used for reducing thread creation time and to better manage the thread life cycle.

We have carried out several load tests and the obtained results have been compared with the sequential reference model. The proposed parallel tumor growth model was tested with a simulation program developed in Java on the Intel Core i7 platform. The simulation program shows a significant improvement with respect to the results of the sequential implementation mentioned in [2].

Our future work is focused on obtaining a new implementation of our tumor growth model for GPU based on the data domain model array with dynamic growth. The proposed model is being improved with the inclusion of dynamic tumor growth characteristics that have been mentioned by various authors [17], such as tumor vascular prominence (angiogenesis) or tumor nutrient intake, which can be modeled using hybrid lattice-gas cellular automata [13].
